# Effect of vitamin A supplementation on gut microbiota in children with autism spectrum disorders - a pilot study

**DOI:** 10.1186/s12866-017-1096-1

**Published:** 2017-09-22

**Authors:** Juan Liu, Xiao Liu, Xue-Qin Xiong, Ting Yang, Ting Cui, Na-Li Hou, Xi Lai, Shu Liu, Min Guo, Xiao-Hua Liang, Qian Cheng, Jie Chen, Ting-Yu Li

**Affiliations:** 1 Department of Child Health Care, Children’s Hospital of Chongqing Medical University, Chongqing, China; 2 Children’s Nutrition Research Center, Children’s Hospital of Chongqing Medical University, No.136 Zhongshan Er Road, Yuzhong District, Chongqing, 400014 People’s Republic of China; 3 Ministry of Education Key Laboratory of Child Development and Disorders, China International Science and Technology Cooperation Base of Child Development and Critical Disorders, Chongqing Key Laboratory of Pediatrics, Chongqing, China; 4 Pediatric Department of Clinical Medicine of Dazhou Vocational and Technical College, Dazhou, Sichuan China

**Keywords:** Autism spectrum disorders, Vitamin A, Gut microbiota

## Abstract

**Background:**

Dysbiosis of gut microbiota are commonly reported in autism spectrum disorder (ASD) and may contribute to behavioral impairment. Vitamin A (VA) plays a role in regulation of gut microbiota. This study was performed to investigate the role of VA in the changes of gut microbiota and changes of autism functions in children with ASD.

**Results:**

Sixty four, aged 1 to 8 years old children with ASD completed a 6-month follow-up study with VA intervention. High-performance liquid chromatography was used to assess plasma retinol levels. The Autism Behaviour Checklist (ABC), Childhood Autism Rating Scale (CARS) and Social Responsiveness Scale (SRS) were used to assess autism symptoms. CD38 and acid-related orphan receptor alpha (RORA) mRNA levels were used to assess autism-related biochemical indicators’ changes. Evaluations of plasma retinol, ABC, CARS, SRS, CD38 and RORA mRNA levels were performed before and after 6 months of intervention in the 64 children. Illumina MiSeq for 16S rRNA genes was used to compare the differences in gut microbiota before and after 6 months of treatment in the subset 20 of the 64 children. After 6 months of intervention, plasma retinol, CD38 and RORA mRNA levels significantly increased (all *P* < 0.05); the scores of ABC, CARS and SRS scales showed no significant differences (all *P* > 0.05) in the 64 children. Meanwhile, the proportion of *Bacteroidetes/Bacteroidales* significantly increased and the proportion of *Bifidobacterium* significantly decreased in the subgroup of 20 (all false discovery rate (FDR) q < 0.05).

**Conclusions:**

*Bacteroidetes/Bacteroidales* were the key taxa related to VA. Moreover, VA played a role in the changes in autism biomarkers. It remains unclear whether the VA concentration is associated with autism symptoms.

**Trial registration:**

The study protocol was peer reviewed and approved by the institutional review board of Children’s Hospital, Chongqing Medical University in 2013 and retrospectively registered in Chinese Clinical Trial Registry (ChiCTR) on November 6, 2014 (TRN: ChiCTR-ROC-14005442).

**Electronic supplementary material:**

The online version of this article (10.1186/s12866-017-1096-1) contains supplementary material, which is available to authorized users.

## Background

Autism spectrum disorder (ASD) is characterized by impaired social interactions and communication, accompanied by repetitive, restricted behavior, interests, or activities. Increasing attention has been paid to the interaction between the gut and the brain in children with ASD. Altered gut microbiota [1–4] are common in children with ASD. Most studies have indicated that gut microbiota changes are strongly correlated with the severity of autism [3, 5], anxiety and depression [6]. Social impairment was observed in germ-free mice [7] and treatment with the probiotic *Bacteroides fragilis* had a beneficial effect on autism behavior in an animal model of autism [8, 9]. The microbiota–gut–brain axis is believed to be involved in the pathogenesis of ASD [10].

Vitamin A deficiency (VAD) is considered a global public health issue, especially in developing countries. Vitamin A (VA) can regulate central nervous system development through its active metabolite retinoic acid (RA). RA is an important factor that promotes intestinal immunity [11] and maintains mucosal epithelial integrity [12]. The gut flora was altered by a VAD diet in rats and mice [13, 14], and RA could restore *Lactobacilli* that were downregulated in a murine lupus model, which was correlated with improved lupus-associated symptoms [15], and provide a reference for the role of VA in systemic lupus erythematosus. These findings indicate that VA also plays a role in the balance of the microbiota barrier.

VAD in consumed food or in the blood of children with ASD compared with typically developing (TD) children or standard values has been reported in several studies [16–18]. In a previous study, we found that deficiencies in ferritin, folate, vitamin B12, 25(OH) vitamin D, and hemoglobin were not as obvious as deficiencies in VA in children with ASD, with the percentage of VAD in ASD as high as 77.9% and the gastrointestinal (GI) symptoms more common in children with ASD than in TD children. Moreover, we found for the first time that the VA levels were negatively correlated with scores of autism symptoms [19].

Therefore, our study aimed to confirm the role of VA on the key gut microbiota and changes in functions in children with ASD.

## Methods

### Participants and procedures

This single-blind, non-randomized intervention pilot study included a total of 64 children with ASD, aged 1 to 8 years old, in 6 special training centers in Chongqing. The study was conducted between August 2013 and April 2015. All parents of the subjects gave their informed consent for inclusion before they participated in the study. All participants were re-diagnosed by two developmental pediatricians in the Children’s Hospital of Chongqing Medical University through a series of structured interviews and met the criteria from the Diagnostic and Statistical Manual of Mental Disorders, 5th edition [20]. The criteria for exclusion were a history of Rett syndrome, cerebral palsy, other congenital diseases, and acute or chronic affective diseases in the recent 3 months, or a usage history of any nutritional supplement or medication in the previous 3 months.

Pediatricians visited the special training centers twice before and after 6 months of vitamin A intervention (VAI). On the first visit, the children were re-diagnosed by two developmental pediatricians. The determined 64 ASD children were evaluated in anthropometric measurements by three professional trained anthropometrists, autism symptoms through the interview with their parents, with the modified version of the Autism Behavior Checklist (ABC), Childhood Autism Rating Scale (CARS) and Social Responsiveness Scale (SRS). They also underwent blood collection for the plasma retinol test and CD38, RORA mRNA levels test, which is used as autism biomarkers. WHO recommended standards for Vitamin A normal (VAN) (> = 1.05 μmol/L), marginal VAD (MVAD) (1.05–0.7 μmol/L), VAD (< 0.7 μmol/L) [21]. VAD is a public health problem in China [22] (http://whqlibdoc.who.int/publications/2009/9789241598019_eng.pdf), where high-dose vitamin A supplementation with 200,000 IU every 4–6 month is recommended in children 12–59 months of age [23] (http://apps.who.int/iris/bitstream/10665/44664/1/9789241501767_eng.pdf?ua=1&ua=1). MVAD may impair lung development [24], learning and memory in children which may result in an enhanced risk of developing Alzheimer’s disease [25, 26]. To avoid impairment in brain and lung development, to satisfy the requirement of WHO for high risk of VAD region, participants with an insufficient plasma retinol status (<1.05 μmol/L) received VAI with a dose of 200,000 IU once orally. It tended to give participants with a sufficient plasma retinol status (> = 1.05 μmol/L) a placebo supplement. All participants enrolled in the study showed MVAD or VAD. Therefore, no placebo group could be formed. All of the evaluations and blood collections were applied again after 6 months of the VAI on the second visit. It tended to give a second VAI for children who still had insufficient VA levels after 6 months of the first VAI on the second visit, but it failed for multiple reasons, details in limitations.

Additionally, fresh stool samples, food frequency questionnaires, mealtime behaviors were asked to be collected from participants who did not receive supplemental probiotics or prebiotics and who were not treated with antibiotics for the previous 1 month in the 64 children before and after the VAI for gut microbiota. But finally a subset of 20 of the 64 children were eligible and agreed to completed the collections for gut microbiota analysis before and after the VAI.

The study protocol was peer reviewed and approved by the institutional review board of Children’s Hospital, Chongqing Medical University in 2013 and registered in Chinese Clinical Trial Registry (ChiCTR) (registration number: ChiCTR-ROC-14005442).

### Measurements

#### Anthropometric measurements

Anthropometric examinations were conducted by three professional trained anthropometrists from Department of Child Health Care of Children’s Hospital, according to standard procedures [27]. Z-scores were calculated for height-for-age (ZHA), weight-for-age (ZWA) and body mass index (BMI; ZBMIA) with WHO Anthro and AnthroPlus software (World Health Organization, 2009, Anthro for Personal Computers, Version 3.01: Software for Assessing Growth and Development of the World’s Children).

#### ASD-related symptoms

SRS has been significantly correlated with the Autism Diagnostic Interview-Revised, with a total score of 60 or higher indicating impairment [28]. CARS is composed of 15 areas, with each area grading from one to four, and a total score of 30 or higher indicating a positive result for autism [29]. The ABC is a scale of 57 different behaviors, and a total score of 53 or higher indicates a probability of childhood autism [30].

#### Food frequency analysis

Only children with ASD who finished the stool sample collection were required to complete the designed food frequency questionnaire, which included 18 types of food. The questionnaire’s options for the frequency of consumption were provided in times per day, per week or per month. All frequencies were converted to times per month for comparison purposes.

### Sample collection and analysis

#### Venous blood sample collection

Two milliliters of venous blood were collected into an ethylene diamine tetraacetic acid anticoagulant tube, transported immediately to the Children’s Hospital within 2 h, then centrifuged at 3000 rpm for 3–5 min to separate the plasma and blood cells. A volume of 200 μL of plasma was stored at −80 °C to measure the retinol concentrations within 1 week. After abundant lysis with red blood cell lysis buffer (TIANGEN, Beijing, China), white cell were separated from whole blood cells. Then the RNA in the white cells was protected in 1 mL of Trizol Reagant (Ambion, Carlsbad, California, USA) at −80 °C for future use.

#### Plasma retinol detection

The plasma retinol levels were tested by high performance liquid chromatography as described in Miller’s study [31].

#### Autism biomarkers detection

CD38 is a multiple-function molecule that might be an early hallmark of autism [32]. CD38 mRNA expression was lower in patients with ASD than in controls, and was correlated with autism symptoms [33]. CD38 SNPs, rs6449197 and rs3796863 were significantly associated with a subset of ASD [34, 35]. Defects in social communication have also been found in CD38 knockout mice [36]. Acid-related orphan receptor alpha (RORA), a transcriptional regulator, has been demonstrated to be a candidate gene of autism [37]. It regulates the A2BP1, CYP19A1, ITPR1, NLGN1, NTRK2 genes [38] and aromatase [39], which may indicate the pathobiology of ASD. Levels of RORA transcription and protein were reduced in ASD compared with controls [40]. RORA was also associated with some symptoms of ASD [39]. These studies indicate that both CD38 and RORA play an important role in ASD. Thus, CD38 and RORA mRNA expression were used as the autism biomarker changes before and after the VAI.

Real-time PCR of CD38 and RORA were used to assess the CD38 and RORA gene expression, following the procedure below. RNA was isolated from the trizol after precipitation, wash and resuspension, in accordance with the Trizol Reagant guidelines (Ambion, Carlsbad, California, USA).Next, the mRNA solution was reverse transcribed into cDNA using the MyCyclerTMThermal Cycler instrument (BIO-RAD, Hercules, California, USA) and PrimeScriptTM RT Master Mix kit (TaKaRa RR036A, DA Lian, Japan). The cDNA quantification by real-time PCR was performed using the CFX ConnectTM Optics Module instrument (BIO-RAD, Hercules, California, USA) and SYBR® Premix Ex TaqTM II(TakaRa RR820A, DA Lian, Japan). Cycling was programmed as 40 cycles of 30 s at 95 °C for initial denaturation, 5 s at 95 °C for denaturation, 30 s at annealing temperature (Table 1) for annealing, and 5 s at 65 °C for extension and a final extension at 95 °C for melt curve analysis. Relative expression levels of CD38 and RORA with β-actin as endogenus reference for mRNA were calculated using gene study section of CFX Manager Software version 2.1 (BIO-RAD, Hercules, California, USA). Primer sequences were designed with the Primer 5 software. The primer was produced by the Beijing Genomics institution. The primer sequences for the PCR genes were listed in Table 1.

**Table 1:** Primer sequences and annealing temperature for the real-time PCR genes

Gene	Primer sequences (5′ to 3′)	Annealing temperature (°C)
CD38	Sense: TTGGGAACTCAGACCGTACCTTG Antisense: CCACACCATGTGAGGTCATC	60.3
RORA	Sense: TCATGGCTGCAAGAAAAGGT Antisense: GAGGAAAATGAAGTCGCACAA	58.3
β-actin	Sense: GTGAAGGTGACAGCAGTCGGTT Antisense: GAGAAGTGGGGTGGCTTTTAGGA	54.7

#### Stool sample collection

Fresh stool samples were collected from participants who did not receive supplemental probiotics or prebiotics and who were not treated with antibiotics for the previous 1 month. The samples were placed into sterile stool tubes, immediately frozen at −20 °C for temporary preservation for less than 7 days, and then transported in a large quantity in a dry ice box to Children’s Hospital within 2 h, where they were frozen at −80 °C for future use.

#### DNA extraction and PCR amplification

Microbial DNA was extracted from the fecal samples (200–250 mg per sample) using the QIAamp® Fast DNA Stool Mini Kit (QIAGEN, Dusseldorf, Germany) according to the manufacturer’s instructions. DNA concentration and purification were determined by NanoDrop 2000 UV-vis spectrophotometer (Thermo Scientific, Wilmington, USA), and DNA quality was checked by 1% agarose gel electrophoresis. The amplification was performed at the Majorbio Bio-Pharm Technology Co., Ltd. (Shanghai, China). The V3-V4 hypervariable regions of the bacterial 16S ribosomal RNA gene was amplified by PCR with thermocycler PCR system (GeneAmp 9700, ABI, USA) in a 20 μL mixture (4 μL of 5 × FastPfu Buffer, 2 μL of 2.5 mM dNTPs, 0.8 μL of forward primer [5 μM], 0.8 μL of reverse primer [5 μM], 0.4 μL of FastPfu Polymerase, 10 ng of template DNA; otherwise, by ddH_2_O)and under the conditions of 3 min of denaturation at 95 °C; 27 cycles of 30 s at 95 °C, 30 s for annealing at 55 °C, and 45 s for elongation at 72 °C; and a final extension at 72 ° for 10 min, using the primers 338F 5′-ACTCCTACGGGAGGCAGCA-3′ and 806R 5′-GGACTACHVGGGTWTCTAAT-3′, where barcode is an eight-base sequence unique to each sample.

#### Illumina MiSeq sequencing

The PCR products were purified using the AxyPrep DNA Gel Extraction Kit (Axygen Biosciences, Union City, California, US) and then quantified using QuantiFluor™-ST (Promega, Madison, US). The amplicons were pooled in equimolar and then paired-end sequenced (2 × 250) on an Illumina MiSeq platform (Illumina, San Diego, USA) according to standard protocols [41] at Majorbio Bio-Pharm Technology Co., Ltd. (Shanghai, China). Raw Illumina data reads were submitted to the NCBI Sequence Read Archive (SRA) database of GenBank under accession ID SRP078095.

#### Bioinformatics analysis

Raw fastq files were demultiplexed, quality-filtered using QIIME (version 1.17) [42] with the following criteria:1) the 250 bp reads were truncated at any site with average quality score < 20 over a 10 bp sliding window and the truncated reads shorter than 50 bp were discarded; 2) exact barcode matches, 2 nucleotide mismatches during primer matching, and reads containing ambiguous characters were removed; and 3) only sequences that overlap longer than 10 bp were assembled according to their overlap sequences, Reads which could not be assembled were discarded.

Operational taxonomic units (OTUs) were chosen using UPARSE (version 7.1 http://drive5.com/uparse/) [43] with a 97% similarity cutoff. Chimeric sequences were detected and removed using UCHIME [44]. The phylogenetic affiliation of each 16S rRNA gene sequence was analyzed by the Ribosomal Database Project Classifier (http://rdp.cme.msu.edu/) against the Silva (SSU119) 16S rRNA database using a confidence threshold of 70% [45].

To standardize the comparisons of the microbiota, to avoid bias in the following analysis, samples were rarefied to 15,673 reads per sample. Community richness (Chao and ACE) and community diversity (Shannon and Simpson) indices were used to evaluate alpha diversity using Mothur [46]. A paired t-test or Wilcoxon’s signed rank test was used for comparisons of the Chao, ACE, Shannon, and Simpson indices between the Pre-VAI and Post-VAI measurements. The Shannon-Wiener curve was analyzed to measure whether the sequencing numbers were reasonable with the use of Mothur [46]. A Specaccum curve was analyzed to measure whether the sample numbers were reasonable [47]. A principal component analysis (PCA) was applied to reflect the differences and distances among the samples in the different groups. Community structure component diagrams were generated to determine the differential trends for the differential taxonomic information of each sample in the different groups. A linear discriminant analysis (LDA) coupled with effect size (LEfSE) [48, 49] was used to find significantly different bacterial taxa between groups at various bacterial levels. LDA scores were used to measure the contribution of each taxon to the significant differences. False discovery rate (FDR) q values were calculated using p.adjust() function in R to correct for multiple testing, with q < 0.05 chosen as the significance threshold.

### Statistical analysis

The means, standard deviations (SDs), medians (P_25_, P_75_), frequencies, and percentages were used to describe all data except for the microbial DNA in our results. The mean ± SD was used for data with a normal distribution, the median (P_25_, P_75_) was used for data with a non-normal distribution, and the frequency (percentage) was used for enumeration data. The two-sample t-test or Wilcoxon’s rank sum test was used to compare the measurement data between two independent samples. χ^2^ test or Fisher’s exact test was used to compare the enumeration data for two independent samples. A paired t-test or Wilcoxon’s signed rank test was used for paired samples.

With the exception of the microbial DNA analysis, the statistical analyses were performed using the SAS software, version 8. Significance was reported at *P* < 0.05.

## Results

**Fig. 1:**
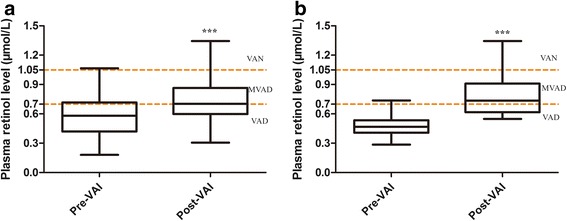
Difference in the plasma retinol level between Pre-VAI and Post-VAI in the total group (*n* = 64) (**a**) and subset (*n* = 20) (**b**). ***P < 0.001, using paired t-tests

**Fig. 2:**
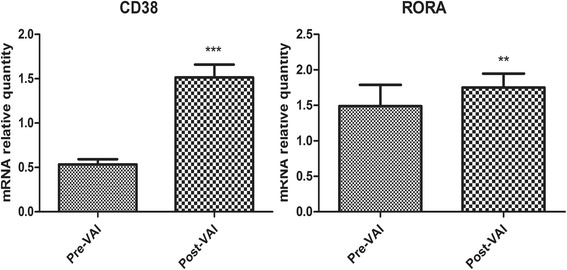
Differences in the CD38 and RORA mRNA levels between Pre-VAI and Post-VAI in the baseline group (*n* = 64). ***P < 0.001, ** *P* < 0.01, using Wilcoxon’s signed rank tests

A total of 64 children with ASD were recruited, and subset of 20 among the 64 children underwent the gut microbiota analysis. As shown in Table 2, the subset of 20 did not significantly differ from the baseline group of 64 in demographic characteristics (eg, gender, age, physical growth levels, autism symptom scale scores, gene expression of autism biomarker CD38 and RORA, parental education, family income). Although the subset significantly differed from total group in plasma retinol level, the significant increases in plasma retinol level after 6 months of the VAI were seen in both groups (Fig. 1). Significant increases in expression of autism biomarker CD38 and RORA after 6 months of the VAI in the baseline group of 64 were also seen in Fig. 2.

**Table 2:** Differences in demographic characteristics between the baseline and subset group

Characteristics	Baseline (*n* = 64)	Subset (*n* = 20)	*P*
Gender and age			
Male, n (%)	55 (86)	17 (85)	0.92^a^
Age (mo.), mean ± SD	62.50 ± 16.34	66.40 ± 13.69	0.29^a^
Age group (years), n (%)			
< 3	3 (5)	0 (0)	0.80^a^
3-	9 (14)	1 (5)	
4-	17 (26)	6 (30)	
5-	19 (30)	7 (35)	
6-	10 (16)	4 (20)	
7–9	6 (9)	2 (10)	
Growth assessment, mean ± SD			
Z_HA_	0.02 ± 1.20	0.33 ± 1.04	0.26^a^
Z_WA_	0.49 ± 1.27	1.06 ± 1.06	0.05^a^
Z_BMIA_	0.70 ± 1.39	1.25 ± 1.23	0.09^a^
Autism symptoms, mean ± SD			
ABC	65.33 ± 21.00	59.80 ± 21.31	0.31^a^
CARS	35.40 ± 9.42	38.07 ± 10.40	0.59^a^
SRS	99.45 ± 26.41	104.4 ± 31.94	0.53^a^
Autism biomarkers, mean ± SD			
CD38/Actin	0.53 ± 0.46	0.43 ± 0.51	0.43^a^
RORA/Actin	1.49 ± 2.39	1.61 ± 2.35	0.84^a^
Plasma retinol level (μmol/L), mean ± SD	0.59 ± 0.19	0.49 ± 0.13	0.01^*^
Father’s educational levels, n (%)			
Middle school or below	23 (36)	6 (30)	0.62^a^
High school	17 (27)	6 (30)	
College or above	24 (37)	8 (40)	
Mother’s educational levels, n (%)			
Middle school or below	18 (28)	5 (25)	0.38^a^
High school	24 (38)	6 (30)	
College or above	22 (34)	9 (45)	
Family annual income (RMB), n (%)			
< 10,000	15 (23)	5 (25)	0.81^a^
10,000–40,000	28 (44)	9 (45)	
> 40,000	21 (33)	6 (30)	

^a^No significant difference was detected using the χ^2^ tests or Fisher’s exact test/two sample t-test; *P < 0.05 using two sample t-test

### Differences in VA levels, autism symptoms and autism biomarkers between pre-VAI and post-VAI in the baseline group (*n* = 64)

The plasma retinol level increased from 0.59 ± 0.19 μmol/L to 0.72 ± 0.20 μmol/L in the group of 64 after 6 months of VA supplementation (Fig. 1a). Percentage of VAN was 0.0% to 3.2%, MVAD was 29.7% to 48.4%, VAD was 70.3% to 48.4%, before and after VAI. Table 3 shows significant differences were not observed in the ABC, CARS and SRS scores between the two groups. Figure 2 revealed that the expression of the ASD-related biomarker CD38 and RORA mRNA increased significantly after the VAI.

**Table 3:** Differences in autism symptoms between Pre-VAI and Post-VAI (*n* = 64)

Symptoms measurements, mean ± SD	Pre-VAI	Post-VAI	*P*
ABC	65.33 ± 21.00	69.48 ± 28.82	0.45^a^
CARS	35.40 ± 9.42	35.51 ± 8.02	0.94^a^
SRS	99.45 ± 26.41	96.14 ± 22.86	0.50^a^

Abbreviation: *ABC* Autism Behavior Checklist, *SRS* Social Responsiveness Scale, *CARS* Childhood Autism Rating Scale; ^a^No significant difference was detected using paired t-tests

### Differences in VA levels, mealtime behaviors, and food frequencies between pre-VAI and post-VAI in the subset group (*n* = 20)

Twenty self-paired stool samples before and after 6 months of VA supplementation were tested for differences in the intestinal microbiota using MiSeq sequencing.

**Fig. 3:**
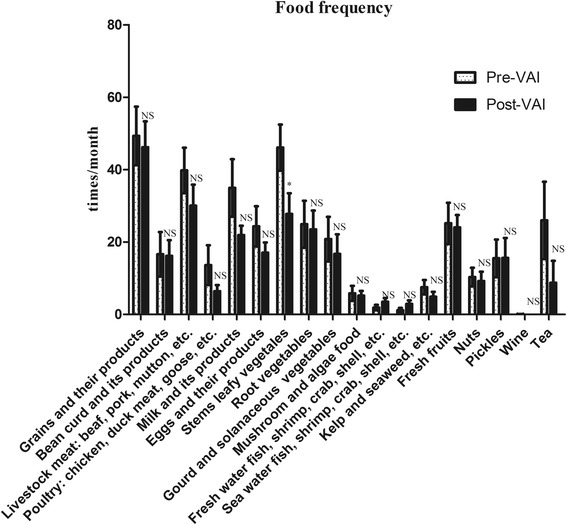
Comparison of food frequencies between Pre-VAI and Post-VAI in the subset group (*n* = 20). The frequencies of each food type were not significantly different between the Pre-VAI and Post-VAI, except for stems and leafy vegetables. * *P* < 0.05, using Wilcoxon’s signed rank tests

The plasma retinol levels increased from 0.49 ± 0.13 μmol/L to 0.78 ± 0.20 μmol/L (Fig. 1b). Table 4 shows that the proportions of picky eating and resistance to new foods behaviors in children with ASD were not significantly different between the Pre-VAI and Post-VAI groups (all *P* > 0.05). The intake frequencies of most food types in all 18 types of foods were not significantly different between the Pre-VAI and Post-VAI groups with the exception of stems and leafy vegetables (Fig. 3). But the FDR q value for stems and leafy vegetables was 0.552, indicating that the significance was false.

**Table 4:** Comparison of mealtime behaviors between the Pre-VAI and Post-VAI groups (*n* = 20)

Mealtime behaviors, n (%)	Pre-VAI	Post-VAI	*P*
Picky eater	12 (60)	16 (80)	0.17^a^
Resistance to new foods	6 (30)	6 (30)	1.00^a^

^a^No significant difference was detected using χ^2^ tests

In summary, the VA levels significantly increased after the VAI in the subset group of 20 subjects. The subjects from the Pre-VAI and Post-VAI groups did not show differences in mealtime behavior and food frequencies which were potential confounding factors that could influence the gut microbiota.

### 16S rRNA deep sequencing for the characterization of the gut microbiota in the subset group

**Fig. 4:**
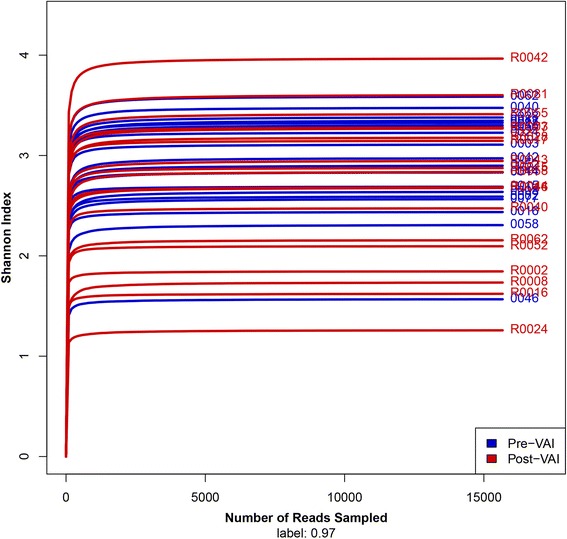
Shannon-Wiener curves

**Fig. 5:**
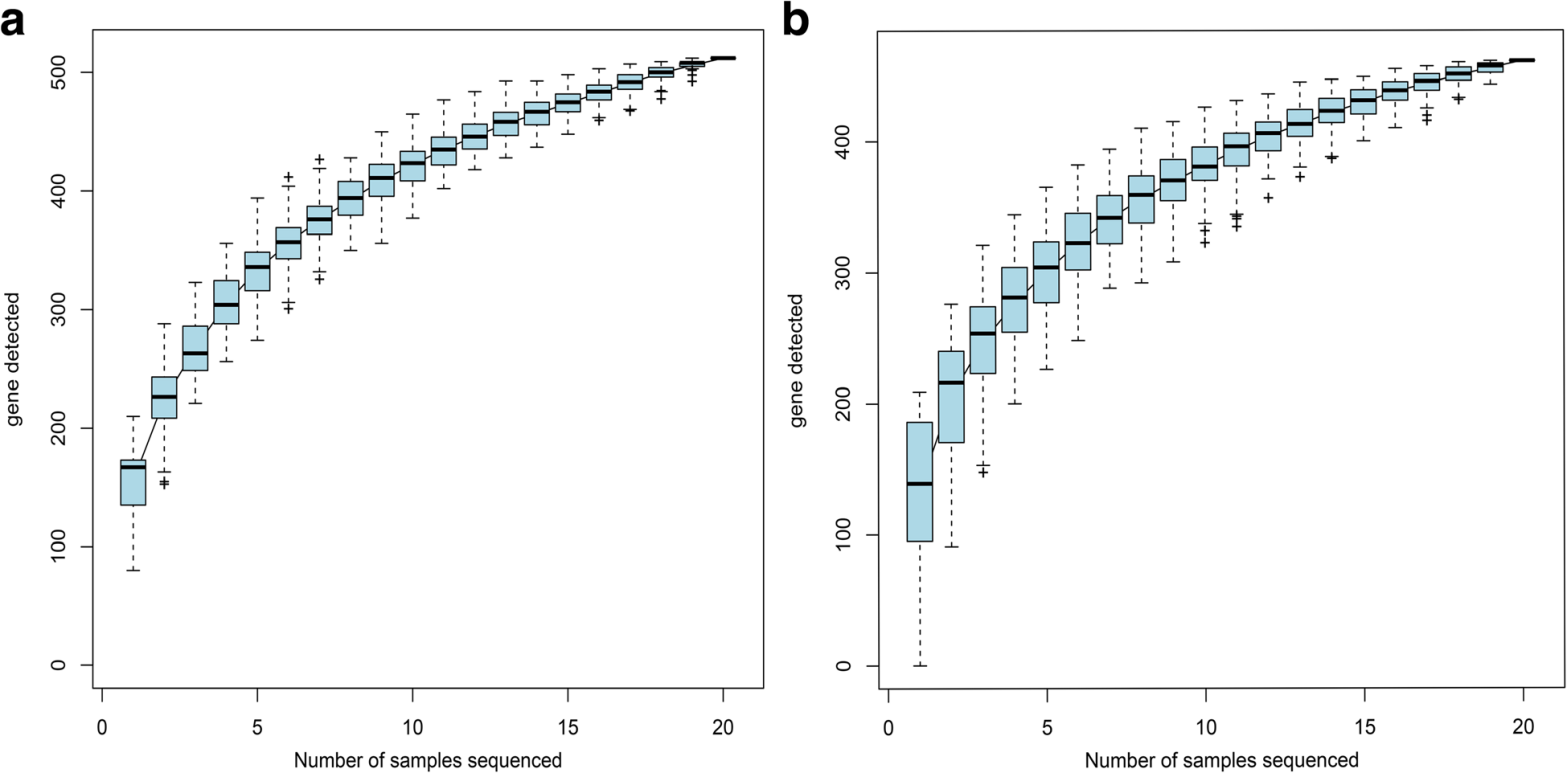
Species accumulation curves. **a** Pre-VAI **b** Post-VAI

After OTU picking and chimera checking, 626,920 (15,673 reads per sample) high quality sequences with an average length of 439.8 bp were obtained in this study. A total of 547 OTUs (similarity level 0.97) and representing16 phyla, 31 classes, 45 orders, 76 families, 148 genera, and 339 species were identified. The Shannon-Wiener curves are shown in Fig. 4. Curve tended to be smooth at or before the 15,673 sequences, indicating that the collected sequences in each sample (15,673) were reasonable. Species accumulation curves are shown in Fig. 5. The OTUs reached a plateau with increases in our samples from the subgroup, indicating that our collected samples in different subgroups were reasonable.

### Changes in the gut microbiota before and after the VAI in the subset group

The differences in the Ace, Chao, Shannon, and Simpson indices between the Pre-VAI and Post-VAI periods were not significant (Table 5).

**Table 5:** Gut microbiota diversity between Pre-VAI and Post-VAI (*n* = 20)

Variables	Pre-VAI	Post-VAI	*P*
ACE, mean ± SD	184.47 ± 34.86	164.79 ± 45.28	0.10^a^
Chao, mean ± SD	181.90 ± 41.10	162.77 ± 46.84	0.12^a^
Shannon, mean ± SD	2.92 ± 0.49	2.69 ± 0.72	0.21^a^
Simpson, median (P25, P75)	0.11 (0.07, 0.15)	0.13 (0.08, 0.22)	0.20^b^

^a^No significant difference was detected using paired t-tests; ^b^No significant difference was detected using Wilcoxon’s signed rank test

**Fig. 6:**
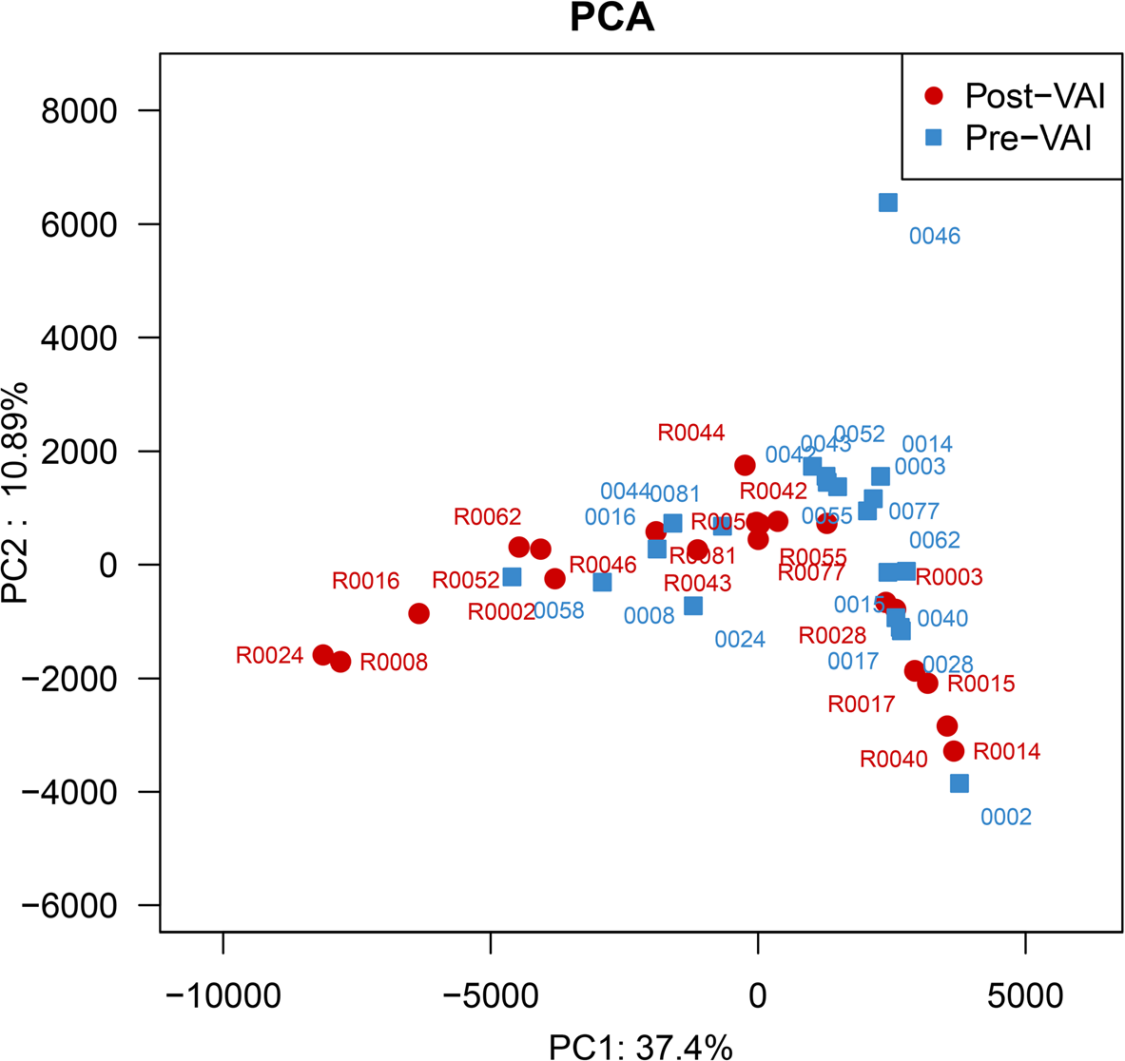
Differences in the bacterial community diversity, richness and structures between the Pre-VAI and Post-VAI groups according to the principal component analysis (PCA). Samples of children with ASD before the VA supplementation (Pre-VAI) (blue dots) were separated from children with ASD after 6 months of VA supplementation (Post-VAI) (red dots)

The location of most Pre-VAI individuals in the PCA decreased after the VAI (Fig. 6) except for 0002-R0002, 0028-R0028, 0044-R0044, 0058-R0058, and 0062-R0062, which indicated that the Pre-VAI individuals separated from the Post-VAI individuals with 37.4% variability on the x-axis and 10.9% variability on the y-axis.

**Fig. 7:**
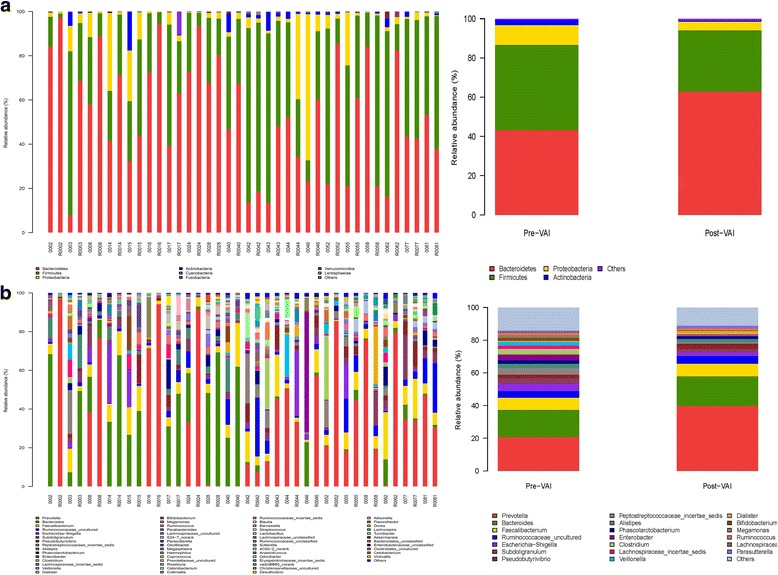
Microbial composition of each sample between Pre-VAI and Post-VAI. **a** *Bacteroidets* (red) tended to increase, and *Firmicutes* (green), *Proteobacteria* (yellow) and *Actinobacteria* (blue) tended to decrease after 6 months of VA supplementation at the phylum level. **b** *Prevotella* (red) and *Bacteroides* (green) increased, and *Bifidobacterium* (brown) *decreased* after 6 months of VA supplementation at the genus level, etc.

The proportion of *Bacteroidetes* in the gut microbiota increased from 43.2% to 62.9% after the VAI, whereas the *Firmicutes* decreased from 43.5% to 31.2% and the ratio of *Bacteroidetes/Fimicutes* increased from 1.0 to 2.0. The proportion of *Proteobacteria* decreased from 10.1% to 4.5%, and *Actinobacteria* decreased from 2.8% to 0.5% at the phylum level in the community structures analysis (Fig. 7a). At the genus level, the proportion of *Prevotella* increased from 20.8% to 40.0%, that of *Bacteroides* increased from 16.6% to 18.2%, that of *Peptostreptococcaceae_incertae_sedis* decreased from 4.2% to 0.9%, that of *Enterobacter* decreased from 3.4% to 0.2%, that of *Escherichia-Shigella* decreased from 4.5% to 2.2%*,* that of *Clostridium* decreased from 3.3% to 0.2%, and that of *Bifidobacterium* decreased from 2.0% to 0.2% after the VAI (Fig. 7b).

**Fig. 8:**
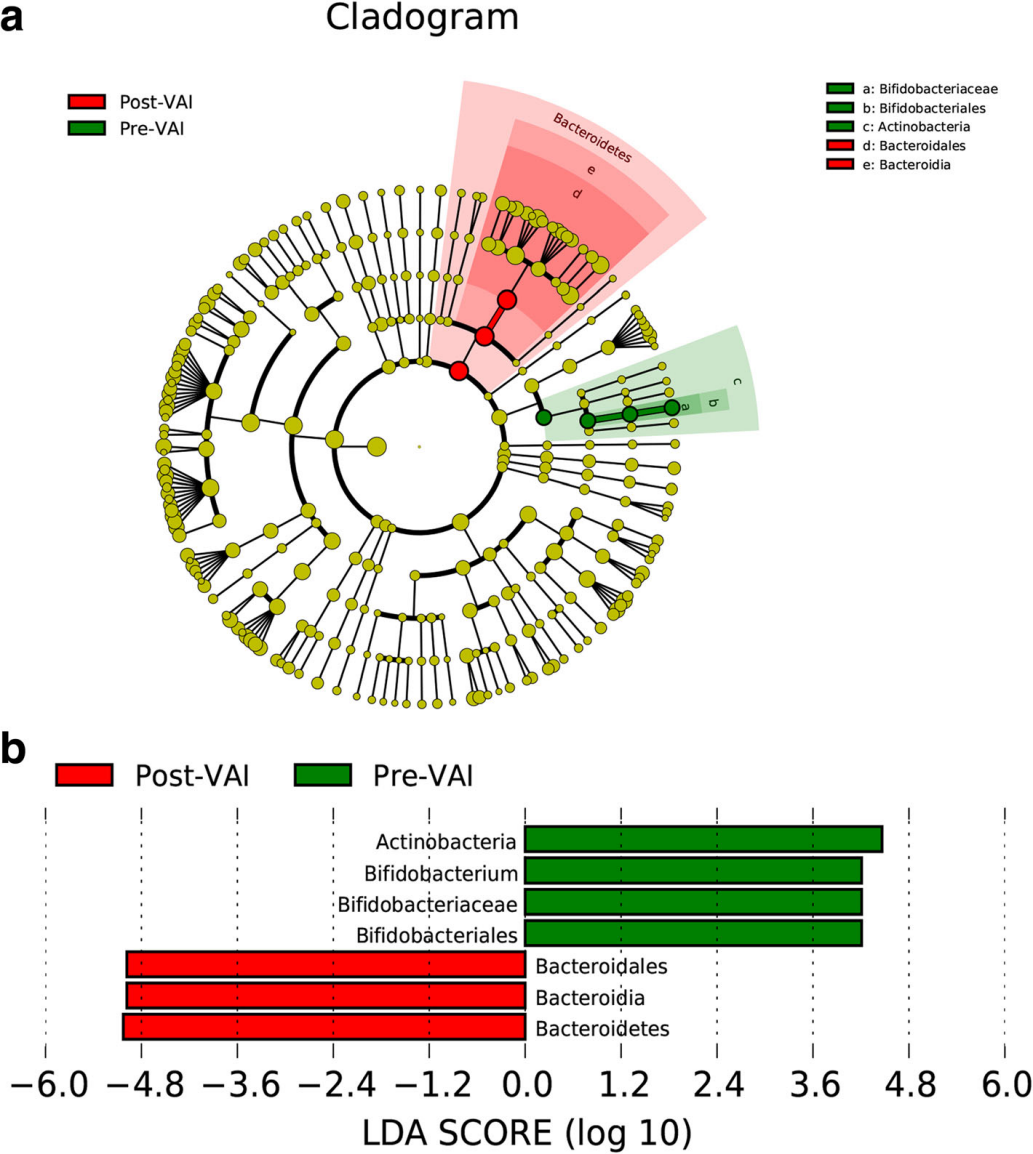
Significant bacterial taxa between subgroups. **a** Cladogram based on the linear discriminant analysis effect size (LEfSE) method, differential feature plots meaning for LEfSe were seen in Additional file 1 and **b** Linear discriminant analysis (LDA) coupled with LEfSE between the Pre-VAI and Post-VAI groups; the results indicated that the taxa Post-VAI were enriched for *Bacteroidetes*, *Bacteroidales,* and *Bacteroidia* (red) compared to those Pre-VAI, while Pre-VAI were enriched for *Bifidobacterium, Bifidobacteriaceae, Bifidobacteriales,* and *Actinobacteria* (green) compared with those Post-VAI

The LEfSE (Fig. 8a and b) indicated that the Post-VAI samples were significantly enriched in *Bacteroidales* at the order level*, Bacteroidia* at the class level, and *Bacteroidetes* at the phylum level compared with the Pre-VAI samples (LDA1 = 5.03, LDA2 = 4.98 and LDA3 = 4.98, respectively; all FDR q = 0.048), whereas the Pre-VAI samples were significantly enriched in *Bifidobacterium, Bifidobacteriaceae* and *Bifidobacteriales* (genus to order) compared with the Post-VAI samples (LDA1 = 4.21, LDA2 = 4.21 and LDA3 = 4.21, respectively; all FDR q = 0.048). Although Pre-VAI were enriched in *Actinobacteria* than Post-VAI in LEfSE (LDA = 4.46), FDR q (0.052) was more than 0.05 after correction for multiple test.

## Discussion

There were significant increases in the proportion of *Bacteroidetes/Bacteroidales* and decreases in *Bifidobacterium* after the VAI, accompanying significant increases in autism biomarkers, while no significant changes were observed in autism symptoms. These findings indicated that VA could regulate gut microbiota and benefit children with ASD partly.

### VA and autism functions

The potential use of VA treatment and the necessity of clinical trials using VA in ASD were illustrated by Megson in 2000 [50]. RA has been considered a transcriptional regulator that can shape synaptic plasticity in the brain, thereby accounting for learning and memory ability [51]. Our previous study found that the cord serum VA level was significantly positively correlated with the language and social area development quotients in children at 2 years of age [52]. Additionally, VA also contributes to the pathogenesis of many neuropsychiatric diseases, such as Alzheimer’s disease [53], multiple sclerosis [54], Parkinson’s disease [55], and fragile X syndrome [56].

CD38 and RORA are critical for society behavior in autism [36, 37, 39]. Interestingly, we found that both CD38 and RORA mRNA levels significantly increased in ASD after the VAI. An in vitro study also demonstrated that RA can upregulate CD38 transcription levels [32, 33]; RA may regulate RORs (including RORA, RORB, RORR) through its retinoic acid receptors [57], which demonstrated the potential role of VA in autism biomarker. However, no significant differences were seen in autism symptoms (ABC, CAR, and SRS scales) before and after the VAI in our study. ASD is a heterogeneous neurodevelopmental disorder and, to date, is generally incurable [58]. A 6-year follow-up study showed that the Vineland Adaptive Behavior Scales scores declined after 6 years of first diagnosis; and most children with ASD exhibited combined moderate-to-severe problems [59]. Thus, ASD intervention is a long persistent, continuous progress. The effect of VA on autism symptoms remains unclear, which deserves a long-time follow-up study to demonstrate.

### VA-related microbiota

Although similar community richness and diversity values were observed in the subgroups, the compositional structure of the gut microbiota differed between the subgroups. To our surprise, after adjusting for mealtime behavior and food intake, the proportion of *Bacteroidetes/Bacteroidales* and the ratio of *Bacteroidetes/Fimicutes* were significantly increased and the proportion of *Bifidobacterium* was significantly decreased after the VAI. *Bacteroidetes/Bacteroidales* and *Bifidobacterium* were perhaps the core microflora related to VA in children with ASD.

Changes in the relative amount of *Bacteroidetes* or *Bacteroides* in children with ASD have been reported in many studies, although the results have differed from study to study. The proportion of *Bacteroidetes* was found at high levels in the ASD [1, 60] in several studies, although it was not significantly different from the control group in another study [61, 62]. Brent L. Williams assessed the microbiota in the ileum of ASD subjects and found that the proportion of *Bacteroidetes* decreased and the *Firmicutes/Bacteroidetes* ratio increased in ASD-GI children; these findings were correlated with the sodium-dependent glucose cotransporter and sucrose isomaltase mRNA levels in the ileum, indicating that the decreased proportion of *Bacteroidetes* in ASD contributed to the impairment of the primary pathway for carbohydrate digestion in ASD [63]. Reductions of the *Bacteroidetes* relative abundance were also seen in Strati’s study [64], Tomova’s study [5]. Another research team supported the application of a *Bacteroides fragilis* probiotic for mental disorders [9]; the results showed that *Bacteroides spp.* application in an autism model ameliorated abnormalities of *Bacteroidales* and improved communicative, repetitive, anxiety-like and sensorimotor behaviors. Additionally, *Bacteroides spp.* restored many serum metabolites related to autism in the model, such as 4-ethylphenylsufate, which is related to the urinary biomarker 4-methylphenol in autism; indolepyruvate, which is a key molecule of the tryptophan metabolism pathway (this finding is reminiscent of the reported increases in inindolyl-3-acryloylglycine in human ASD); serotonin, which is another key factor for the tryptophan metabolism pathway; and hyperserotonemia, which has been observed in human ASD [8]. Besides, a meta-analysis showed lower level of *Bacteroides* is associated with inflammatory bowel disease [65]. *Bacteroides fragilis* is critical for the normal function of the mammalian immune system [66]. The upregulation of VA in *Bacteroidetes* was demonstrated by Liu in a mouse study. RA reduced the ratio of *Firmicutes* to *Bacteroidetes*, which was associated with accelerated liver regeneration [67].

The proportion of *Bifidobacterium* was reportedly lower in ASD [1, 68], The bacteria acts as a type of probiotic, promoting the production of different exopolysaccharides that act as fermentable substrates for different gut bacteria [69]. *Bifidobacterium* is correlated with phenol and short and medium chain fatty acids, which are metabolites via the gut bacteria and are closely tied to ASD [1]. Nevertheless, our results showed that the proportion of *Bifidobacterium* decreased after the VAI. Another study performed probiotic supplementation in children with autism and showed that the *Bacteroidetes/Firmicutes* ratio in the children’s feces was normalized and that the relative amount of *Bifidobacterium* had significantly decreased [5], which was similar to the findings in our study. The decrease in the *Bifidobacterium*, a common probiotic, might have contributed to the increase in the proportion of dominant bacteria of *Bacteroidetes*, or might be a VAI-related effect, or other reasons, which needs further study to explain.

In summary, many researches with high impact factor voiced that *Bacteroidetes/Bacteroides* and *Bacteroidetes/Firmicutes* ratio was reduced in ASD; *Bacteroides* intervention may improve social behaviors in autism. After VAI, this key beneficial microbiota increased, along with increases in autism-related CD38 and RORA mRNA levels. Thus, we assumed that VA might have some benefits on autism by upregulating the proportion of *Bacteroidetes* or the *Bacteroidetes/Firmicutes* ratio in human ASD, which requires further study for confirmation.

### Limitations

The following are limitations of our study. First, most children still had MVAD or VAD at 6 months in our participants, suggesting that they should have accepted a second VAI with 200,000 IU, according to WHO suggestion [23]. In fact, it tended to give VA supplementation twice a year, but ASD participants failed to follow-up for multiple reasons, such as remove, parental divorce, economic reasons, other drugs or nutrients supplements influence, unwilling to accept the invasive examination or oral VA intervention again, etc. These reasons, together with the strict request for fresh stool collection and restoration, contributed to the second limitation that total sample size of 64 and the subset size of 20 for the gut microbiota analysis were relatively small. Though the low samples size for the gut microbiota, it was powered for high quality controls: without use with probiotics, prebiotics, antibiotics, other drugs or nutrients supplements in ASD participants; without significant difference in mealtime behaviors and food frequencies in the Pre-VAI and Post-VAI. Besides, species accumulation curves were also applied and finally indicated the rationality of our subgroup samples. Third, we aimed to conduct a placebo-controlled intervention study, but all the participants showed an insufficient VA status and were thus enrolled into the VAI group.

## Conclusions

VA may promote changes in the gut microbiota composition and have some benefits in the improvement of autism biomarkers CD38 and RORA in children with ASD. *Bacteroidetes/Bacteroidales* were the key taxa related to VA in children with ASD.

Future studies are necessary to address how VA affects the gut microbiota and the exact function of the VA-related microbiota in autism, especially the relationship among the VA-related microbiota and autism biomarkers CD38 and RORA. It remains unclear whether VA status is associated with autism symptoms, which requires further investigation to demonstrate.

## Additional file

Additional file 1: Differential feature plots meaning for LEfSe. (DOCX 11 kb)

## Abbreviations

ABC: Autism behavior checklist
ASD: Autism spectrum disorder
CARS: Childhood autism rating scale
FDR: False discovery rate
GI: Gastrointestinal
LDA: Linear discriminant analysis
LEfSE: Linear discriminant analysis effectsize
MVAD: Marginal vitamin A deficiency
OTU: Operational taxonomic unit
PCA: Principal component analysis
RA: Retinoic acid
RORA: Acid-related orphan receptor alpha
SRS: Social responsiveness Scale
TD: Typically developing
VA: Vitamin A
VAD: Vitamin A deficiency
VAI: Vitamin A intervention
ZBMIA: Z-scores for body mass index
ZHA: Z-scores for height-for-age
ZWA: Z-scores for weight-for-age

## References

[1] De Angelis M, Piccolo M, Vannini L, Siragusa S, De Giacomo A, Serrazzanetti DI (2013). Fecal microbiota and metabolome of children with autism and pervasive developmental disorder not otherwise specified. PLoS One.

[2] Kang DW, Park JG, Ilhan ZE, Wallstrom G, Labaer J, Adams JB (2013). Reduced incidence of Prevotella and other fermenters in intestinal microflora of autistic children. PLoS One.

[3] Adams JB, Johansen LJ, Powell LD, Quig D, Rubin RA (2011). Gastrointestinal flora and gastrointestinal status in children with autism--comparisons to typical children and correlation with autism severity. BMC Gastroenterol.

[4] Parracho HM, Bingham MO, Gibson GR, McCartney AL (2005). Differences between the gut microflora of children with autistic spectrum disorders and that of healthy children. J Med Microbiol.

[5] Tomova A, Husarova V, Lakatosova S, Bakos J, Vlkova B, Babinska K (2015). Gastrointestinal microbiota in children with autism in Slovakia. Physiol Behav.

[6] Foster JA, McVey Neufeld KA (2013). Gut-brain axis: how the microbiome influences anxiety and depression. Trends Neurosci.

[7] Desbonnet L, Clarke G, Shanahan F, Dinan TG, Cryan JF (2014). Microbiota is essential for social development in the mouse. Mol Psychiatry.

[8] Hsiao EY, McBride SW, Hsien S, Sharon G, Hyde ER, McCue T (2013). Microbiota modulate behavioral and physiological abnormalities associated with neurodevelopmental disorders. Cell.

[9] Gilbert JA, Krajmalnik-Brown R, Porazinska DL, Weiss SJ, Knight R (2013). Toward effective probiotics for autism and other neurodevelopmental disorders. Cell.

[10] Cryan JF, Dinan TG (2012). Mind-altering microorganisms: the impact of the gut microbiota on brain and behaviour. Nat Rev Neurosci.

[11] Cassani B, Villablanca EJ, De Calisto J, Wang S, Mora JR (2012). Vitamin a and immune regulation: role of retinoic acid in gut-associated dendritic cell education, immune protection and tolerance. Mol Asp Med.

[12] McCullough FS, Northrop-Clewes CA, Thurnham DI (1999). The effect of vitamin a on epithelial integrity. Proc Nutr Soc.

[13] Amit-Romach E, Uni Z, Cheled S, Berkovich Z, Reifen R (2009). Bacterial population and innate immunity-related genes in rat gastrointestinal tract are altered by vitamin A-deficient diet. J Nutr Biochem.

[14] Cha HR, Chang SY, Chang JH, Kim JO, Yang JY, Kim CH (2010). Downregulation of Th17 cells in the small intestine by disruption of gut flora in the absence of retinoic acid. J Immunol.

[15] Zhang H, Liao X, Sparks JB, Luo XM (2014). Dynamics of gut microbiota in autoimmune lupus. Appl Environ Microbiol.

[16] Hyman SL, Stewart PA, Schmidt B, Cain U, Lemcke N, Foley JT (2012). Nutrient intake from food in children with autism. Pediatrics.

[17] Sun C, Xia W, Zhao Y, Li N, Zhao D, Wu L (2013). Nutritional status survey of children with autism and typically developing children aged 4-6 years in Heilongjiang Province, China. J Nutr Sci.

[18] Shabayek MM (2004). Assessment of the nutritional status of children with special needs in Alexandria: I. Nutrient intake and food consumption. J Egypt Public Health Assoc.

[19] Liu X, Liu J, Xiong X, Yang T, Hou N, Liang X, et al. Correlation between Nutrition and Symptoms: Nutritional Survey of Children with Autism Spectrum Disorder in Chongqing, China. Nutrients. 2016;8(5). doi:10.3390/nu8050294. 10.3390/nu8050294 PMC4882707 27187463

[20] Nemeroff CB, Weinberger D, Rutter M, MacMillan HL, Bryant RA, Wessely S (2013). DSM-5: a collection of psychiatrist views on the changes, controversies, and future directions. BMC Med.

[21] Jiang W, Yu Q, Gong M, Chen L, Wen EY, Bi Y (2012). Vitamin a deficiency impairs postnatal cognitive function via inhibition of neuronal calcium excitability in hippocampus. J Neurochem.

[22] WHO (2009). Global prevalence of vitamin a deficiency in populations at risk 1995–2005. WHO global database on vitamin a deficiency.

[23] WHO (2011). Guildeline:vitamin a supplementation in infants and children 6–59 months of age.

[24] Wei H, Huang HM, Li TY, Qu P, Liu YX, Chen J (2009). Marginal vitamin a deficiency affects lung maturation in rats from prenatal to adult stage. J Nutr Sci Vitaminol (Tokyo).

[25] Zeng J, Chen L, Wang Z, Chen Q, Fan Z, Jiang H (2017). Marginal vitamin a deficiency facilitates Alzheimer's pathogenesis. Acta Neuropathol.

[26] Zeng J, Li T, Gong M, Jiang W, Yang T, Chen J (2017). Marginal vitamin a deficiency exacerbates memory deficits following Abeta1-42 injection in rats. Curr Alzheimer Res.

[27] de Onis M, Onyango AW, Van den Broeck J, Chumlea WC, Martorell R (2004). Measurement and standardization protocols for anthropometry used in the construction of a new international growth reference. Food Nutr Bull.

[28] Alabdali A, Al-Ayadhi L, El-Ansary A (2014). A key role for an impaired detoxification mechanism in the etiology and severity of autism spectrum disorders. Behav Brain Funct.

[29] Rapin I, Goldman S (2008). The Brazilian CARS: a standardized screening tool for autism. J Pediatr.

[30] Marteleto MR, Pedromonico MR (2005). Validity of autism behavior checklist (ABC): preliminary study. Rev Bras Psiquiatr.

[31] Miller KW, Yang CS (1985). An isocratic high-performance liquid chromatography method for the simultaneous analysis of plasma retinol, alpha-tocopherol, and various carotenoids. Anal Biochem.

[32] Ebstein RP, Mankuta D, Yirmiya N, Malavasi F (2011). Are retinoids potential therapeutic agents in disorders of social cognition including autism?. FEBS Lett.

[33] Riebold M, Mankuta D, Lerer E, Israel S, Zhong S, Nemanov L (2011). All-trans retinoic acid upregulates reduced CD38 transcription in lymphoblastoid cell lines from autism spectrum disorder. Mol Med.

[34] Higashida H, Munesue T (2013). CD38 and autism spectrum disorders. No To Hattatsu.

[35] Munesue T, Yokoyama S, Nakamura K, Anitha A, Yamada K, Hayashi K (2010). Two genetic variants of CD38 in subjects with autism spectrum disorder and controls. Neurosci Res.

[36] Jin D, Liu HX, Hirai H, Torashima T, Nagai T, Lopatina O (2007). CD38 is critical for social behaviour by regulating oxytocin secretion. Nature.

[37] Hu VW (2012). Is retinoic acid-related orphan receptor-alpha (RORA) a target for gene-environment interactions contributing to autism?. Neurotoxicology.

[38] Sarachana T, Hu VW (2013). Genome-wide identification of transcriptional targets of RORA reveals direct regulation of multiple genes associated with autism spectrum disorder. Mol Autism.

[39] Sarachana T, Xu M, Wu RC, Hu VW (2011). Sex hormones in autism: androgens and estrogens differentially and reciprocally regulate RORA, a novel candidate gene for autism. PLoS One.

[40] Nguyen A, Rauch TA, Pfeifer GP, Hu VW (2010). Global methylation profiling of lymphoblastoid cell lines reveals epigenetic contributions to autism spectrum disorders and a novel autism candidate gene, RORA, whose protein product is reduced in autistic brain. FASEB J.

[41] Caporaso JG, Lauber CL, Walters WA, Berg-Lyons D, Huntley J, Fierer N (2012). Ultra-high-throughput microbial community analysis on the Illumina HiSeq and MiSeq platforms. ISME J.

[42] Caporaso JG, Kuczynski J, Stombaugh J, Bittinger K, Bushman FD, Costello EK (2010). QIIME allows analysis of high-throughput community sequencing data. Nat Methods.

[43] Edgar RC (2013). UPARSE: highly accurate OTU sequences from microbial amplicon reads. Nat Methods.

[44] Edgar RC, Haas BJ, Clemente JC, Quince C, Knight R (2011). UCHIME improves sensitivity and speed of chimera detection. Bioinformatics.

[45] Amato KR, Yeoman CJ, Kent A, Righini N, Carbonero F, Estrada A (2013). Habitat degradation impacts black howler monkey (Alouatta Pigra) gastrointestinal microbiomes. ISME J.

[46] Schloss PD, Gevers D, Westcott SL (2011). Reducing the effects of PCR amplification and sequencing artifacts on 16S rRNA-based studies. PLoS One.

[47] Maughan H, Wang PW, Diaz Caballero J, Fung P, Gong Y, Donaldson SL (2012). Analysis of the cystic fibrosis lung microbiota via serial Illumina sequencing of bacterial 16S rRNA hypervariable regions. PLoS One.

[48] Segata N, Izard J, Waldron L, Gevers D, Miropolsky L, Garrett WS (2011). Metagenomic biomarker discovery and explanation. Genome Biol.

[49] Zhang C, Li S, Yang L, Huang P, Li W, Wang S (2013). Structural modulation of gut microbiota in life-long calorie-restricted mice. Nat Commun.

[50] Megson MN (2000). Is autism a G-alpha protein defect reversible with natural vitamin a?. Med Hypotheses.

[51] Shearer KD, Stoney PN, Morgan PJ, McCaffery PJ (2012). A vitamin for the brain. Trends Neurosci.

[52] Zhang X, Chen K, Wei XP, Qu P, Liu YX, Chen J (2009). Perinatal vitamin a status in relation to neurodevelopmental outcome at two years of age. Int J Vitam Nutr Res.

[53] Sodhi RK, Singh N (2014). Retinoids as potential targets for Alzheimer's disease. Pharmacol Biochem Behav.

[54] Fragoso YD, Stoney PN, McCaffery PJ (2014). The evidence for a beneficial role of vitamin a in multiple sclerosis. CNS Drugs.

[55] Takeda A, Nyssen OP, Syed A, Jansen E, Bueno-de-Mesquita B, Gallo V (2014). Vitamin a and carotenoids and the risk of Parkinson's disease: a systematic review and meta-analysis. Neuroepidemiology.

[56] Soden ME, Chen L (2010). Fragile X protein FMRP is required for homeostatic plasticity and regulation of synaptic strength by retinoic acid. J Neurosci.

[57] Stehlin-Gaon C, Willmann D, Zeyer D, Sanglier S, Van Dorsselaer A, Renaud JP (2003). All-trans retinoic acid is a ligand for the orphan nuclear receptor ROR beta. Nat Struct Biol.

[58] Bolte S (2014). Is autism curable?. Dev Med Child Neurol.

[59] Olsson MB, Westerlund J, Lundstrom S, Giacobini M, Fernell E, Gillberg C (2015). "recovery" from the diagnosis of autism - and then?. Neuropsychiatr Dis Treat.

[60] Finegold SM, Dowd SE, Gontcharova V, Liu C, Henley KE, Wolcott RD (2010). Pyrosequencing study of fecal microflora of autistic and control children. Anaerobe.

[61] Gondalia SV, Palombo EA, Knowles SR, Cox SB, Meyer D, Austin DW (2012). Molecular characterisation of gastrointestinal microbiota of children with autism (with and without gastrointestinal dysfunction) and their neurotypical siblings. Autism Res.

[62] Son JS, Zheng LJ, Rowehl LM, Tian X, Zhang Y, Zhu W (2015). Comparison of fecal microbiota in children with autism Spectrum disorders and Neurotypical siblings in the Simons simplex collection. PLoS One.

[63] Williams BL, Hornig M, Buie T, Bauman ML, Cho Paik M, Wick I (2011). Impaired carbohydrate digestion and transport and mucosal dysbiosis in the intestines of children with autism and gastrointestinal disturbances. PLoS One.

[64] Strati F, Cavalieri D, Albanese D, De Felice C, Donati C, Hayek J (2017). New evidences on the altered gut microbiota in autism spectrum disorders. Microbiome.

[65] Zhou Y, Zhi F (2016). Lower level of Bacteroides in the gut microbiota is associated with inflammatory bowel disease: a meta-analysis. Biomed Res Int.

[66] Troy EB, Kasper DL (2010). Beneficial effects of Bacteroides Fragilis polysaccharides on the immune system. Front Biosci (Landmark Ed).

[67] Liu HX, Hu Y, Wan YJ (2016). Microbiota and bile acid profiles in retinoic acid-primed mice that exhibit accelerated liver regeneration. Oncotarget.

[68] Wang L, Christophersen CT, Sorich MJ, Gerber JP, Angley MT, Conlon MA (2011). Low relative abundances of the mucolytic bacterium Akkermansia muciniphila and Bifidobacterium spp. in feces of children with autism. Appl Environ Microbiol.

[69] Salazar N, Gueimonde M, Hernandez-Barranco AM, Ruas-Madiedo P (2008). De los Reyes-Gavilan CG. Exopolysaccharides produced by intestinal Bifidobacterium strains act as fermentable substrates for human intestinal bacteria. Appl Environ Microbiol.

