# Supplementary material for: Effect of vitamin A supplementation on gut microbiota in children with autism spectrum disorders - a pilot study
Source: BMC Microbiol. 2017 Sep 22;17:204. doi: 10.1186/s12866-017-1096-1 (PMC5610466; doi:10.1186/s12866-017-1096-1)
Supplement: Additional file 1: — Differential feature plots meaning for LEfSe. (DOCX 11 kb) [file 12866_2017_1096_MOESM1_ESM.docx]

Cladogram based on the linear discriminant analysis effect size (LEfSE) method in figure8: yellow dots indicate no significant differences between the two groups; red indicates phylotypes that were statistically overrepresented in Post-VAI compared to Pre-VAI, and green indicates phylotypes that were overrepresented in Pre-VAI compared to Post-VAI.
